# Determinants of implementation success for a digital single-session intervention for workplace mental health: Mixed methods evaluation in a cluster trial

**DOI:** 10.1016/j.invent.2026.100970

**Published:** 2026-06-23

**Authors:** Philip J. Batterham, Amelia Gulliver, Cassandra Heffernan, Alison L. Calear, Aliza Werner-Seidler, Alyna Turner, Louise M. Farrer, Michael Berk

**Affiliations:** a Centre for Mental Health Research, The Australian National University, Australia; b Black Dog Institute, University of New South Wales, Australia; c Deakin University, IMPACT – the Institute for Mental and Physical Health and Clinical Translation, School of Medicine, Faculty of Health and Barwon Health, Australia; d School of Public Health and Preventive Medicine, Monash University, Australia; e Orygen, The National Centre of Excellence in Youth Mental Health, Centre for Youth Mental Health, Florey Institute for Neuroscience and Mental Health, Department of Psychiatry, The University of Melbourne, Melbourne, Australia

**Keywords:** Implementation, Uptake, Acceptability, Service use, Mental health, Workplace, Employee, Internet

## Abstract

**Background:**

Programs to support mental health in workplaces, particularly those using digital methods, show promise for increasing appropriate use of services. However, it is unclear which factors influence the successful implementation of such programs.

**Methods:**

Implementation outcomes from a cluster randomised trial (cRCT) of a digital single-session intervention (Helipad program) for improving mental health help seeking in employees were explored using both qualitative interviews (*n* = 16) and quantitative data collected at post-test during the cRCT (*N* = 344). Linear regression analyses tested predictors of participation (uptake) by workplace, and predictors of participant views about the acceptability, appropriateness, and feasibility of both the Helipad program and the control (static psychoeducation only). Qualitative data were thematically analysed to summarise feedback and recommendations around implementing the program, based on the Consolidated Framework for Implementation Research.

**Results:**

The Helipad program performed better than the control program for participant implementation outcomes, with significantly superior acceptability, appropriateness, and feasibility. More intensive methods to encourage participation such as additional emails, meetings, and delivery via a learning management system were associated with significantly greater uptake. There were few differences in acceptability ratings between workplace types indicating that the program may be useful and acceptable in a broad range of settings. Qualitative data indicated that the Helipad program was easy-to-use and contained a broad range of information on workplace mental health. A challenge identified by participants was how to best engage people with less interest or low perceived need for mental health support. Recommendations for implementation included ensuring workplace leader investment, allowing time during work to complete the program and modelling positive attitudes and behaviours towards mental health and help seeking.

**Conclusions:**

Implementation of workplace mental health interventions can be enhanced by clear communication with workplace leaders about its benefits, and by facilitating engagement of employees with the program during work time. Partnerships with stakeholders such as insurance companies or industry bodies may improve reach.

**Trial registration**: Australian New Zealand Clinical Trials Registry (ANZCTR) ACTRN12623000270617.

## Highlights

• Digital workplace interventions may increase appropriate use of mental health services.
• We tested implementation outcomes in a cluster RCT of an internet intervention.
• The co-designed active intervention had better implementation outcomes than an active control.
• Qualitative interviews indicated workplace leadership and facilitated engagement are critical for implementation.
• Multi-layered mental health promotion may support to mentally healthy workplaces.

## 1 Introduction

A high proportion of working-aged adults (16–65 years) experience mental disorders. They account for over 80% of the global burden of disease from poor mental health (Santomauro et al., 2026). In Australia, anxiety disorders (17%) and depression (5%) are most prevalent (Australian Bureau of Statistics, 2023). However, fewer than half of adults with clinical symptoms access professional services (Australian Bureau of Statistics, 2023). Inadequate mental health support in the workplace can compromise employee health and lead to reductions in functioning, quality of life, and productivity, as well as difficulties in interpersonal relationships, and occupational burnout (Bouwmans et al., 2014; Lee et al., 2017; Schofield et al., 2019; Bagasi et al., 2025; ten Have et al., 2013; de Oliveira et al., 2023). Poor mental health can also lead to additional costs, with chronic mental health problems a leading cause of workplace disability (Kalski et al., 2025). Therefore, preventing or reducing the burden of poor mental health in the workplace and ensuring those who experience these conditions receive timely support is critical to improving health and economic outcomes. However, there remain gaps in the evidence base for digital interventions to promote service use, and critically, limited evidence around strategies to implement effective interventions into workplaces.

Workplace programs can increase employees' access to appropriate mental health support (Fukita et al., 2025). However, there is limited evidence about what strategies are most effective in facilitating the implementation of workplace mental health interventions. A recent scoping review by Bernard et al. (2022) explored implementation strategies for digital workplace mental health and wellbeing programs, finding that implementation data was either piecemeal or not reported at all (Bernard et al., 2022). Implementation factors were broadly mapped to the RE-AIM domains (Glasgow et al., 1999) with common strategies including encouragement via mass communication via email, the provision of information during meetings and promotional material, and providing incentives (Reach), measuring user experience throughout program implementation (Effectiveness), educational meetings with decision-makers (Adoption), surveys, reviews, and process evaluations (Implementation), and accommodating work conditions and embedding programs within existing structures (Maintenance) (Bernard et al., 2022). However, there was little information on which were most effective (Bernard et al., 2022). In addition, this study also examined implementation barriers and enablers specifically in the context of digital mental health programs. A total of 217 barrier and facilitator measures were identified by Bernard et al. (2022) using the Consolidated Framework for Implementation Research (Damschroder et al., 2009). These included both inner (e.g., resources such as time, physical space, and support to use the program, multiple media formats to support accessibility), and outer context (e.g., external policy perceived to potentially breach confidentiality), and individual user characteristics (e.g., attitudes, motivation, digital skills) (Bernard et al., 2022). Two systematic reviews and meta-syntheses (Paterson et al., 2024; Yarker et al., 2022) exploring broader (i.e., not only digital) mental health program implementation identified similar barriers, including a lack of prioritisation or support from management, low organisational resources, and perceived stigma and lack of privacy. Enablers included tailored content, leadership buy-in and support, leaders' knowledge of mental health problems, allowing employees to complete programs in work hours, and flexible delivery times (Paterson et al., 2024; Yarker et al., 2022). However, factors influencing engagement are likely to differ in face-to-face interventions, and very few of the included studies specifically targeted help seeking.

Few studies have evaluated mental health interventions delivered in the workplace that aim to improve help seeking or service use. Fewer still have explored factors impacting their implementation (Hanisch et al., 2016; Ramírez-Vielma et al., 2023). Cornish et al. (2019) tested an intervention for military personnel, finding participants who had higher distress and self-stigma had poorer engagement. Griffiths et al. (2016) found managerial staff engaged significantly less with an online program than non-managerial staff. Billings et al. (2008) showed that an online work mental health promotion program was acceptable to employees. While there is limited research on strategies to engage employees in digital mental health programs, research on workplace physical activity interventions suggests that active buy-in from employers and flexible enrolment strategies may be critical to encourage engagement (Mullane et al., 2019; Ryde et al., 2013). However, there remain few rigorous studies examining a range of factors that influence the success of implementing digital help-seeking programs into workplace settings.

We sought to address this gap by exploring implementation outcomes in the context of a hybrid type 1 cluster randomised controlled trial (cRCT) of a co-designed online workplace program for improving help seeking for mental health problems (Helipad). We based our evaluation on the Consolidated Framework for Implementation Research, with the *individuals* domain (individual characteristics that influence implementation) and *implementation process* domain (uptake and engagement) evaluated based on quantitative data from the trial to identify individual differences in implementation outcomes and the effectiveness of different strategies to encourage uptake. Qualitative interview data was then used to examine the roles of all five domains: *individuals*, *implementation process*, *innovation* (characteristics of the intervention), *inner setting* (team and leadership), and *outer setting* (broader workplace context).

## 2 Methods

### 2.1 Aim

Our aim was to identify factors associated with better implementation outcomes at the individual level (feasibility, acceptability, appropriateness) and the workplace level (uptake), including the roles of intervention condition, participant characteristics and workplace characteristics. In a subsample of intervention group participants, we aimed to qualitatively explore factors that were considered important to the implementation of workplace mental health interventions.

### 2.2 Trial design

We used a two-arm cRCT to assess the effectiveness and implementation of the Helipad program compared with standard psychoeducation alone on improving workplace employees' help-seeking intentions. Previous publications detail in full the development of the program (Gulliver et al., 2025), protocol and full trial methodology (Batterham et al., 2024), and effectiveness outcomes of the trial, which demonstrated superiority of the Helipad program relative to control based on the primary endpoint (Batterham et al., n.d.). Additional file 1 presents the CONSORT checklist (Hopewell et al., 2025).

### 2.3 Procedure

Two custom-designed websites were created with integrated assessments (self-report pre-test, post-test, and 6-month follow-up), together with the two online programs described below, and with programmable email reminders to participants. Participants were recruited via workplaces (clusters) into the trial, which was conducted online. Only data from pre-test, post-test and qualitative interviews contributed to the current analyses.

#### 2.3.1 Interventions

##### 2.3.1.1 The Helipad program

The programs have been described in detail previously (Gulliver et al., 2025; Batterham et al., n.d.). Briefly, the fully automated online Helipad program comprised five interactive modules completed in a single session lasting around 15–30 min. Content was divided into the five key topics of 1) Recognising symptoms, 2) Getting support, 3) Treatment options, 4) Helping others, and 5) Supportive workplaces. Modules included interactive tools and quizzes, videos, printable lists, and links to external webpages on mental health, targeting mental health literacy (knowledge about common mental health problems such as depression and anxiety), stigmatising attitudes and concerns about workplace disclosure, information about types of professionals available [e.g., GP, psychologist, Employee Assistance Program (EAP)], and how to support those with mental ill-health in the workplace.

##### 2.3.1.2 Active control program

The control program was time-matched and included five modules containing standard written psychoeducation on mental health and wellbeing derived from the National Institutes of Health (NIH) News in Health newsletters (newsinhealth.nih.gov) (Farrer et al., 2024). Modules were not interactive and included basic information on: 1) Sleep and wellbeing, 2) Exercise and physical health, 3) Mental health, 4) Social relationships and health, and 5) Stress and health. We expected the control program to be a robust comparator as it was likely to improve mental health literacy and stigma, but less likely to impact our primary outcome of help-seeking intentions (Corrigan et al., 2012).

#### 2.3.2 Workplace recruitment

We recruited workplaces using a variety of methods, including direct approaches such as targeted phone calls, emails and messaging and online paid and unpaid advertising via social media platforms, LinkedIn and Sales Navigator. Our advertisements emphasised the costs of untreated mental ill-health, and benefits for employees and workplaces (see Additional file 2 for examples). Interested workplaces provided a signed copy of a form confirming agreement to all aspects of the trial, and that their workplace met the inclusion criteria:

1) 50+ employees i.e., medium-to-large workplaces.
2) EAP available to ensure staff could seek mental health support if required.

No restrictions were placed on the industry or the location of the workplace within Australia.

#### 2.3.3 Randomisation

We used minimisation (Treasure and MacRae, 1998) to randomly allocate workplaces to either the (1) Helipad program or (2) the active control condition (see (Batterham et al., n.d.) for details). Workplaces were balanced across conditions on the following characteristics: 1) workplace type (office-based vs. non-office based); 2) workplace size (<200 vs. 200+); and location (ACT/NSW/VIC vs. Other Australian state/territory).

#### 2.3.4 Participant recruitment

Because we aimed to increase the ecological validity of the study and gather information on implementation, workplaces were provided with a customisable invitation email to distribute to their employees. Additional file 2 includes a typical email invitation. This email included an embedded link to their assigned study recruitment website, which provided written information about the study and included a downloadable PDF including this information. Nearly half (*n* = 13) of the 32 sites (*n* = 17) altered the email, and three workplaces (7 sites) elected to send the invitation in a different form (e.g., emailed staff newsletter). Customised changes to text typically included organisational endorsement of the study, encouragement for employees to participate, or further context to ensure the email was not perceived as junk mail. In addition, key representatives from one large workplaces (*n* = 5 sites) worked with the research team, their IT department and head office over several months to directly embed invitations to the study into their customised workplace learning platform. No incentives or payments for participation were provided to either workplaces or individuals, except for participation in qualitative interviews (AU$50 e-gift card). Participant inclusion criteria included being an employee in a participating Australian workplace, aged 18 years or over, living in Australia, fluent in reading and understanding English, and with access to a device and a reliable internet connection.

#### 2.3.5 Participants and data collection

Data on workplace characteristics were collected during recruitment. Individual participant data was collected at the pre-test, immediate post-test and 6-month surveys. We obtained process data on recruitment methods used by the workplaces during the trial. These methods were categorised based on the intensity of encouragement: 1) Minimal (e.g., standard email or staff newsletter and 1-week reminder with minimal tailoring), 2) Some (e.g., 1–2 additional methods to increase participation such as encouragement and motivation of staff and workplace leaders via staff meetings, team chats, or reminder emails), and 3) Comprehensive (e.g., embedded program in learning management system + additional reminders and emails).

The trial flow chart is presented in Additional file 3. Of the 614 employees (interventio*n* = 282; control = 332) who consented to participate, *N* = 487 (intervention = 223, 79.1%; control = 264, 79.5%) participated in the trial. A total of 344 people (interventio*n* = 141, 50.0%; control = 203, 60.3%) completed the post-test implementation measures and were included in the current study. We also invited participants in the active intervention to participate in a follow-up interview to discuss their experiences and insights on implementation, with 16 participants completing a separate informed consent process and participating in the interviews.

#### 2.3.6 Measures

##### 2.3.6.1 Implementation measures: acceptability, appropriateness, feasibility

At the post-test assessment participants completed three validated 4-item measures (Weiner et al., 2017) to assess the acceptability (Acceptability of Intervention Measure, AIM), appropriateness (Intervention Appropriateness Measure, IAM), and the feasibility (Feasibility of Intervention Measure, FIM) of the intervention. A mean score was calculated for each scale with lower scores on each indicating a lack of acceptability, appropriateness, or feasibility. All three measures have been validated (Weiner et al., 2017) and in the current study, internal consistency (Cronbach's α) was 0.92 for AIM, and 0.96 for IAM, and 0.94 for FIM.

##### 2.3.6.2 Demographic characteristics

Data on demographic characteristics collected at pre-test included: gender, age, language spoken at home, ethnicity, level of education, employment status, and work industry. All demographic survey items included a ‘prefer not to answer’ option. Given the complex nature of ethnicity in Australia (Stevens and Fozdar, 2021), participants were able to use open ended text to self-identify, which were then coded into Australian ethnicity classifications (Australian Bureau of Statistics, 2011), and categorised into the most prevalent categories: ‘Australian’, ‘North-West European/Caucasian’, ‘Asian’, and ‘Other.’

##### 2.3.6.3 Mental health measures

Mental illness stigma was measured using a bespoke scale created by using four items from the ‘stigma to others’ scale from the Stigma and Self-Stigma Scales (SASS) (Docksey et al., 2022). Items were rated on a 5-point scale of agreement (0–4); higher mean scores indicate greater stigma (SASS, α = 0.81). We measured mental health literacy using 10 items from the Depression (D-Lit, (Griffiths et al., 2004)) and Anxiety Literacy (A-Lit) questionnaires (Gulliver et al., 2012) and one new item: “Avoiding professional help for my mental health is unlikely to have an effect on my health long-term” (false). Eleven statements were rated true/false/don't know, with correct answers scoring 1 point (range 0–11; higher scores = higher literacy) (Griffiths et al., 2004; Gulliver et al., 2012). Help-seeking intentions were measured using the General Help-seeking Questionnaire (GHSQ) intentions scale (Wilson et al., 2005), based on intentions to seek help from professional sources (mean of “psychologist or counsellor”, “psychiatrist”, and “family doctor/GP”); (GHSQ α = 0.79). General psychological distress was assessed using the 5-item Distress Questionnaire (DQ5) (Batterham et al., 2016). Items were rated based on frequency over the past 30 days (never = 1 to always = 5); total scores range from 5 to 25, with higher scores indicating greater levels of psychological distress (α = 0.87).

#### 2.3.7 Follow-up qualitative interviews

Interviews explored experiences using the program and ideas to facilitate implementation using open-ended questions. A full list of the interview questions is presented in Additional file 4. From a pool of *n* = 40 participants who indicated willingness to be interviewed at post-test, 16 interviews were completed a median of 14 days after post-test. The 16 participants were selected on the basis of diverse experiences, work levels (e.g., management, employees), ages, and gender, with a maximum of two interviewees per workplace. The interview was recorded on Zoom videoconference software or audio recorded over telephone for accuracy and transcription. References to participants' names or workplaces were removed in transcription to reduce the possibility of identification. Interview participants were offered an AU$50 e-gift card as remuneration.

#### 2.3.8 Deviations from protocol

As described in the primary outcomes paper (Gulliver et al., 2025), there were some unexpected deviations from the protocol, although none related to this evaluation of implementation outcomes. After the allocation of 20 organizations, we noticed that the automated minimization programming failed to account for stratification of factors, which was rectified by switching to manual minimization performed by an independent staff member. A planned update to the online platform at the end of February 2025 briefly disrupted the 6-month reminders, and there were minor errors in sending invitation emails during the trial that did not reach participants.

### 2.4 Outcomes and data analysis

Our protocol details the original study aims, from which investigation of implementation factors was an exploratory aim (see Aim 3 in (Batterham et al., 2024)); thus we had no specific hypotheses. In addition, given we co-designed the intervention to appeal to a broad workplace audience, we hypothesised no substantial differences in implementation outcomes based on demographic or workplace factors, but aimed to test this assumption explicitly. For the quantitative data, between-condition differences and participant/workplace characteristics and recruitment methods associated with better implementation outcomes at post-test were individually examined using descriptive statistics (*t*-tests) and linear regression. A linear regression analysis was used to examine predictors of employee participation rates (uptake) by workplace, with all assumptions met. For the qualitative data, thematic analysis (Braun and Clarke, 2006) was used to extract key feedback on the program and suggestions for implementing the program. An initial summary of the interview data was conducted (CH), followed by classification of this data into basic codes (AG) and adding exemplar quotes for each code. Codes were then summarised (AG) into overarching themes, based on interpretation of the quote's meaning (Braun and Clarke, 2021), which were mapped onto the five CFIR implementation domains (*innovation, outer setting, inner setting, individuals, implementation process*) (Damschroder et al., 2022). The CFIR *individuals* domain was also addressed based on the quantitative data.

## 3 Results

Table 1 presents demographic and mental health data for participants completing the post-test survey implementation measures (*N* = 344). Table 2 presents workplace characteristics (*N* = 51) and workplace reported methods for increasing program ‘uptake’ or participation in the study.

**Table 1:** Demographic information for participants (*N* = 344) included in the study.

	Control		Helipad		Total	
Participant characteristics	(*n* = 203)		(*n* = 141)		(*N* = 344)	
	*n*	(%)	*n*	(%)	*n*	(%)
Age category (years)						
18–25	19	(9.4)	14	(9.9)	33	(9.6)
26–35	57	(28.1)	42	(29.8)	99	(28.8)
36–45	54	(26.6)	36	(25.5)	90	(26.2)
46–55	43	(21.2)	24	(17.0)	67	(19.5)
56–65	24	(11.8)	22	(15.6)	46	(13.4)
66+	5	(2.5)	3	(2.1)	8	(2.3)
Prefer not to answer	1	(0.5)	14	(9.9)	1	(0.3)
Gender						
Male	44	(21.7)	34	(24.1)	78	(22.7)
Female	157	(77.3)	106	(75.2)	263	(76.5)
Non-binary	1	(0.5)	1	(0.7)	2	(0.6)
Prefer not to answer	1	(0.5)	0	(0.0)	1	(0.3)
Language spoken at home						
English only	162	(79.8)	100	(70.9)	262	(76.2)
English and another language	40	(19.7)	39	(27.7)	79	(23.0)
Another language only	1	(0.5)	2	(1.4)	3	(0.9)
Ethnicity						
Australian	79	(38.9)	37	(26.2)	116	(33.7)
North-West European/Caucasian	73	(36.0)	62	(44.0)	135	(39.2)
Asian	25	(12.3)	22	(15.6)	47	(13.7)
Other	26	(12.8)	20	(14.2)	46	(13.4)
Highest level of education						
High school or less	14	(6.9)	4	(2.8)	18	(5.2)
Certificate/diploma	57	(28.1)	26	(18.4)	83	(24.1)
Bachelor's degree	62	(30.5)	55	(39.0)	117	(34.0)
Postgraduate degree/diploma	69	(34.0)	54	(38.3)	123	(35.8)
Prefer not to answer	1	(0.5)	2	(1.4)	3	(0.9)
Employment						
Full-time	160	(78.8)	111	(78.7)	271	(78.8)
Part-time/casual	42	(20.7)	30	(21.3)	72	(20.9)
Not working (e.g., study, maternity leave)	1	(0.5)	0	(0.0)	1	(0.3)
Industry						
Public services or administration	84	(41.4)	45	(31.9)	129	(37.5)
Health or social care	46	(22.7)	27	(19.1)	73	(21.2)
Teacher training or education	18	(8.9)	19	(13.5)	37	(10.8)
Science or pharmaceuticals	20	(9.9)	3	(2.1)	23	(6.7)
Recruitment or HR	13	(6.4)	6	(4.3)	19	(5.5)
Engineering or manufacturing	2	(1.0)	10	(7.1)	12	(3.5)
Environment or agriculture	2	(1.0)	7	(5.0)	9	(2.6)
Hospitality or events	0	(0.0)	9	(6.4)	9	(2.6)
Business, consultancy or management	5	(2.5)	2	(1.4)	7	(2.0)
Computing or IT	2	(1.0)	3	(2.1)	5	(1.5)
Transport or logistics	3	(1.5)	1	(0.7)	4	(1.2)
Other	8	(4.0)	9	(6.4)	17	(4.9)
Mental health measures	***M***	***SD***	***M***	***SD***	***M***	***SD***
Mental illness stigma (SASS) (mean, range 0–4)	2.57	(2.51)	2.68	(2.73)	2.62	(2.61)
Mental health literacy (Combined D-lit/A-lit) (total range 011)	7.63	(2.06)	7.35	(2.21)	7.50	(2.13)
General psychological distress (DQ5) (total, range 5–25)	11.04	(4.54)	11.14	(4.05)	11.09	(4.32)
Help-seeking intentions (GHSQ, professionals –*M* score of psychologist or counsellor, psychiatrist, GP (*M* range 1–7))	3.89	(1.53)	3.78	(1.42)	3.84	(1.48)

Note: *n = 2 workplaces (both medium, NSW/VIC/ACT, Metropolitan, Control condition) had 0 participants completing the post-test implementation measures.

**Table 2:** Characteristics of workplaces (*n* = 51).

	Control		Helipad		Total	
Workplace characteristics	(*n* = 26)*		(*n* = 25)		(*n* = 51)	
	*n*	(%)	*n*	(%)	*n*	(%)
Workplace type						
Non-office	11	(42.3)	10	(40.0)	21	(41.2)
Mostly office-based	15	(57.7)	15	(60.0)	30	(58.8)
Organisation size						
Medium <200	18	(69.2)	17	(68.0)	35	(68.8)
Large ≥200	8	(30.8)	8	(32.0)	16	(31.4)
State/territory						
NSW/VIC/ACT	20	(76.9)	21	(84.0)	41	(80.4)
Other	6	(23.1)	4	(16.0)	10	(19.6)
Location						
Metropolitan	22	(84.6)	22	(88.0)	44	(86.3)
Regional	4	(15.4)	3	(12.0)	7	(13.7)
Workplace methods to increase uptake						
*Minimal* (standard email or newsletter).	15	(57.7)	13	(52.0)	28	(54.9)
*Some* (1–2 additional methods i.e., pre-emptive email, mentioned in meeting)	8	(30.8)	10	(40.0)	18	(35.3)
*Comprehensive* (embedded in learning management system + multiple methods, reminders)	3	(11.5)	2	(8.0)	5	(9.8)

### 3.1 Quantitative correlates of better implementation outcomes: individuals CFIR domain

Table 3 presents the quantitative data on acceptability, appropriateness, and feasibility collected immediately after program completion. All three were rated significantly higher on average by participants in the intervention group compared with the control.

**Table 3:** Comparison between conditions on post-test measures for participants included in the study (*n* = 344).

	Program				Test of significance			
	Control		Active					
Factor	*n*	*Mean (SD)*	*n*	*Mean (SD)*	*t*	*df*	*p*	*d*
Acceptability (AIM)	203	3.64 (0.76)	141	3.90 (0.71)	3.10	342	**.002**	0.34
Appropriateness (IAM)	202	3.66 (0.77)	140	3.87 (0.71)	2.54	340	**.011**	0.28
Feasibility (FIM)	202	3.84 (0.67)	140	4.11 (0.59)	3.83	340	**<.001**	0.42

Workplace participation rates across both conditions ranged from 0.0% to 26.9% (*M* = 6.03, *SD* = 5.46). The regression analysis showed that compared with minimal encouragement (e.g., standard emails or newsletter alone), some encouragement (*B* = 4.48, *SE* = 1.57, *t* = 2.86, *p* = .006) and comprehensive encouragement (*B* = 5.31, *SE* = 2.52, *t* = 2.11, *p* = .041), were significant predictors of increased participation rates. No other workplace characteristics significantly influenced participation. Additional file 5 presents the full data for the linear regression model.

Table 4 presents linear regressions for predictors of acceptability, appropriateness and feasibility, by condition.

**Table 4:** Linear regression models for implementation outcomes predicted by demographic, personal and workplace data (*N* = 344).

	*Acceptability*				*Appropriateness*				*Feasibility*			
	*Estimate*	*SE*	*t*	*p*	*Estimate*	*SE*	*t*	*p*	*Estimate*	*SE*	*t*	*p*
Active program												
Model summary	*R*^*2*^ = 0.172; *Adjusted R*^*2*^ = 0.071; *SE* = 0.681				*R*^*2*^ = 0.158; *Adjusted R*^*2*^ = 0.054; *SE* = 0.673				*R*^*2*^ = 0.182; *Adjusted R*^*2*^ = 0.081; *SE* = 0.569			
Intercept	4.429	0.465	9.524	<.001	4.32	0.46	9.399	<.001	4.101	0.389	10.533	<.001
Gender (male)	−0.053	0.144	−0.372	.710	−0.022	0.142	−0.156	.876	0.014	0.120	0.117	.907
**Age**^b^ *18–35 years*	−0.212	0.187	−1.132	.260	−0.267	0.187	−1.428	.156	0.075	0.156	0.477	.634
*36–55 years*	−0.362	0.176	−2.061	**.041**^a^	−0.311	0.173	−1.792	.076	0.007	0.147	0.047	.962
**Education**^c^ *Cert/diploma/bachelor degree*	−0.653	0.302	−2.165	**.032**^a^	−0.367	0.298	−1.229	.221	−0.388	0.252	−1.540	.126
*Higher degree*	−0.835	0.318	−2.631	**.010**^a^*	−0.599	0.314	−1.904	.059	−0.360	0.265	−1.357	.177
Employment (full-time)	0.192	0.162	1.190	.236	−0.02	0.160	−0.125	.901	0.106	0.135	0.782	.436
Language (English only)	0.202	0.151	1.342	.182	0.139	0.149	0.932	.353	0.137	0.127	1.085	.280
Workplace type (non-office)	0.238	0.143	1.659	.100	0.301	0.142	2.127	**.035**^a^	0.242	0.120	2.014	**.046**^a^
Workplace size (medium 〈200)	0.056	0.151	0.373	.710	−0.065	0.150	−0.436	.664	0.122	0.127	0.960	.339
State/territory (NSW/VIC/ACT)	0.203	0.162	1.250	.214	0.296	0.160	1.843	.068	0.318	0.136	2.345	**.021**^a^
Location (metropolitan)	0.041	0.180	0.228	.820	−0.087	0.178	−0.489	.626	0.180	0.151	1.194	.235
Help-seeking intentions	0.025	0.045	0.571	.569	0.011	0.044	0.252	.802	0.038	0.038	1.017	.311
Stigma	−0.023	0.031	−0.729	.467	−0.028	0.031	−0.914	.363	−0.034	0.026	−1.312	.192
Mental health literacy	0.043	0.033	1.318	.190	0.032	0.032	0.987	.326	0.023	0.027	0.824	.412
General psychological distress	−0.025	0.016	−1.579	.117	−0.021	0.016	−1.331	.186	−0.028	0.013	−2.083	**.039**^a^


	*Acceptability*				*Appropriateness*				*Feasibility*			
	*Estimate*	*SE*	*t*	*p*	*Estimate*	*SE*	*t*	*p*	*Estimate*	*SE*	*t*	*p*
Control program												
Model summary	*R*^*2*^ = 0.081; *Adjusted R*^*2*^ = 0.006; *SE* = 0.761				*R*^*2*^ = 0.085; *Adjusted R*^*2*^ = 0.011; *SE* = 0.773				*R*^*2*^ = 0.098; *Adjusted R*^*2*^ = 0.025; *SE* = 0.669			
Intercept	3.215	0.435	7.387	<.001	3.439	0.443	7.768	<.001	3.540	0.383	9.254	<.001
Gender (male)	0.056	0.135	0.417	.677	0.054	0.137	0.397	.692	0.094	0.119	0.795	.428
**Age** *18–35 years*	−0.118	0.185	−0.640	.523	−0.210	0.188	−1.117	.266	−0.329	0.166	−1.988	**.048**^a^
*36–55 years*	−0.219	0.175	−1.257	.210	−0.258	0.178	−1.455	.147	−0.386	0.157	−2.463	**.015**^a^
**Education** *Cert/diploma/bachelor degree*	0.248	0.225	1.106	.270	0.216	0.228	0.947	.345	0.152	0.198	0.767	.444
*Higher degree*	0.068	0.239	0.283	.778	0.000	0.243	−0.001	.999	0.064	0.210	0.303	.762
Employment (Full-time)	0.019	0.141	0.133	.895	0.005	0.143	0.032	.975	−0.090	0.124	−0.728	.468
Language (English only)	0.036	0.143	0.249	.804	0.046	0.145	0.317	.752	0.102	0.126	0.809	.420
Workplace type (non-office)	−0.047	0.146	−0.324	.746	−0.081	0.149	−0.546	.586	0.081	0.128	0.631	.529
Workplace size (medium <200)	−0.062	0.112	−0.553	.581	−0.063	0.114	−0.554	.580	−0.022	0.098	−0.227	.821
State/territory (NSW/VIC/ACT)	0.221	0.173	1.274	.204	0.249	0.176	1.416	.158	0.184	0.152	1.21	.228
Location (metropolitan)	−0.378	0.224	−1.687	.093	−0.476	0.229	−2.077	**.039**^a^	−0.330	0.199	−1.655	.100
Help-seeking intentions	0.074	0.040	1.851	.066	0.055	0.040	1.369	.173	0.041	0.035	1.156	.249
Stigma	−0.018	0.027	−0.657	.512	−0.015	0.028	−0.529	.598	−0.030	0.024	−1.235	.219
Mental health literacy	0.009	0.031	0.294	.769	0.015	0.032	0.491	.624	0.011	0.028	0.385	.700
General psychological distress	0.009	0.013	0.648	.518	0.003	0.014	0.237	.813	0.018	0.012	1.479	.141

a ***p*** **<** **.05.** Reference group is presented in (brackets).

b (vs. 56+ years).

c (vs. high school or less).

#### 3.1.1 Acceptability

The acceptability of the active program (Helipad) was significantly related to age and education levels. Acceptability was lower in those aged 36–55 years than among older adults (56+ years). Those with mid-range education levels (certificate/diploma or bachelor's degree) and those with post-graduate qualifications reported lower levels of acceptability than those with lower education completion levels. No factors were significantly related to acceptability for the control program.

#### 3.1.2 Appropriateness

Views on appropriateness of the Helipad program were significantly related to workplace type, with those working in office-based settings reporting higher appropriateness ratings than those in non-office settings. For the control program, working in a regional area was associated with lower ratings on appropriateness compared to working in a metropolitan area.

#### 3.1.3 Feasibility

Living in an area outside our primary catchment and working in a mostly-office based workplace (vs non-office settings) were associated with higher feasibility ratings for the active Helipad program. Those with greater levels of distress at pre-test rated Helipad lower on feasibility than those with lower levels of distress. Finally, for the control program, only age was related to feasibility, with those aged 18–55 years reporting lower perceived feasibility than older adults.

### 3.2 Barriers and enablers of implementation identified in qualitative interviews

There were 11 women and 5 men (total *n* = 16) from the Helipad condition who participated in an interview. Participants had a mean age of 42.7 years (*SD* = 12.7; range = 23–67 years) and described working in a variety of industries including education (*n* = 4), public service (*n* = 4) agriculture (*n* = 2), manufacturing (*n* = 2), health, recreation, research, and technology (*n* = 1 each). There was a mix of non-managerial (*n* = 12) and managerial or hiring roles (*n* = 4).

#### 3.2.1 Innovation CFIR domain

Feedback on the Helipad program (i.e., the innovation (Damschroder et al., 2022)) was primarily related to content and functionality, including its appearance. The strengths of Helipad's content included its informative, holistic, and well-rounded approach to mental health in workplaces and that it would likely improve awareness, particularly in those who had limited knowledge of mental health. However, this was noted as an important but difficult to achieve implementation goal to engage this group in workplaces.

*You don't really engage with it unless you need to, or unless you have some kind of connection to it. I think the challenge is really around…awareness raising amongst those people who haven't had anything to do with this kind of space.*

*(Female, 26–35 years, employee, public services)*

One aspect that was observed by several participants was that Helipad successfully demystified mental health and the process of professional help-seeking and treatment.

*You actually explain what it is and what to expect, and how a session is likely to work and so, you're destigmatising and demystifying that experience, and, I haven't seen that anywhere else and I think there's a bit of power in it.”*

*(Male, 56–65 years, manager, manufacturing)*

Another participant emphasised the utility of the program in reducing stigma and ensuring people understand that *“mental health can impact people's life and work, and [it's] not that I'm not a good person, it's actually I'm sick and…recognise that it is ok to seek help, it's not being weak.” (Female, 36–45 years, employee, technology).* A detailed summary of feedback on the content and functionality, including on specific modules is presented in Additional file 6.

#### 3.2.2 Individuals CFIR domain

Supplementing the individual drivers of implementation outcomes identified from the quantitative data, interview participants reported varying motivations to try the Helipad program. These included that it was an alternative to underutilised EAPs, *“I don't think we're getting what we need from [the EAP]” (Male, 56–65 years, manager, manufacturing)* or because staff were reluctant to use EAPs because of concerns they are too *“light on”* or their *“privacy isn't respected” (Female, 46–55 years, employee, education).* Others were motivated to use Helipad because of an existing interest in mental health and wellbeing, alignment with their current workplace values, they were encouraged by leadership in their workplaces, and to build skills, with one noting it's *“not just about finding out where you are. It's about learning and developing your skill, giving you resources to further your own understanding of mental health” (Female, 46–55 years, employee, agriculture)*. Engagement with the program was also motivated by wanting to complete it once started – *“I just started it and then I thought I should finish it.” (Male, 46–55 years, employee, education).*

#### 3.2.3 Implementation process domain

##### 3.2.3.1 Engaging leadership

Many of the participants remarked that support and promotion by leaders within their employer, such as HR or in conjunction with support by CEO/senior leadership would increase involvement in the Helipad program. This was consistent with our quantitative findings, where additional leadership encouragement was associated with higher uptake rates. Several participants indicated that this support was the main reason they participated in the trial: *“it means that there's something that they may or may not get out of it and why not support that” (Male, 26–35 years, employee, manufacturing).* Gaining traction with key area heads such as managers and supervisors was expressed as being important. Encouragement from leaders in the workplace such as being *“strongly recommended by the workplace” (Male, 26–35 years, employee, manufacturing)* was also broadly viewed by participants in employee roles as being likely to increase worker engagement.

##### 3.2.3.2 Engaging employees

Some suggestions for physical reminders to increase engagement included displaying materials in the workplace. For example, one participant in a management position described how their staff were initially concerned that the invitation email was not legitimate, so they put together a poster instead to increase uptake of the intervention.

*Organic engagement, as people are walking into a lunchroom…They just scan the QR code and go and do it…Just trying to find different ways to get the message out…not everyone reads an email or whatever, just to get it in their face a little bit more.*

*(Female, 36–45 years, manager, agriculture)*

To maximise impact, one manager pointed out that displaying images from the program around the workplace may reinforce and enable ongoing access to key points after program completion. For example, the traffic light model of mental health from the program could be printed on *“a little poster or something…[to] help that self-awareness and self-assessment on a daily work basis.” (Female, 36–45 years, manager, agriculture).*

Another suggestion for engaging employees was to appeal to altruism in the workplace. One participant said, *“I think it's a really good resource for potentially someone who is supporting someone going through mental health struggles.” (Female, 26–35 years, employee, public services).* Making the program mandatory was suggested by several participants, both managers:*“I think it has to be made part of the onboarding process” (Female, 36–45 years, manager, education*) and employees, as a way of increasing engagement in workplaces: *“I feel like this would be such a good thing to incorporate into [mandatory training]” (Female, 26–35 years, employee, public services)*. It was perceived as important for managers to complete the program in addition to employees, to increase *“emotional intelligence”* and promote *“openness” (Female, 46–55 years, manager, recreation)* and sufficient knowledge on how to recognise and support mental health in the workplace.

Irrespective of whether the program was compulsory, embedding it into existing learning management systems was likely to be useful to increase accessibility and engagement: *“we could put it in the LMS and assign it to different skills…something that you can do as part of your development.” (Female, 36–45 years, manager, agriculture)*. This perspective was also reflected in the quantitative finding that use of an LMS was associated with increased participation rates.

#### 3.2.4 Inner setting domain

As for the Implementation process domain, leadership that endorses and encourages participation in workplace programs was seen as critical for uptake. Again, this was consistent with our finding of higher uptake among workplaces that provided more methods of encouragement. Positive workplace culture and attitudes towards mental health were viewed as another important setting factor, promoting conversations about mental health that may reduce stigma and support program uptake – *“every time we talk about this, we're reducing stigma. You know, you're making it more and more normal” (Female, 36–45 years, manager, education).*

Employees feeling they have sufficient time to give it *“the attention it might deserve” (Female, 46–55 years, employee, education)*, and managers communicating and allowing this time for staff to complete the program during work hours was considered important. For example, managers might encourage employees to: *“take an hour out of your workday to participate in this because we think it's beneficial” (Female, 26–35 years, employee, public services).* Adequate time was also viewed as critical because it meant staff were aware they had *“permission”* to complete the program during work hours *(Female, 36–45 years, manager, agriculture).* Additionally, participants thought it was important for management to speak positively and offer encouragement to use the program, such as learning new skills, where a *“small investment now could make you feel better in the future.” (Male, 56–65 years, employee, education)*.

#### 3.2.5 Outer setting domain

Demonstrating the program's importance to key workplace leaders, along with human resources or health and safety teams, was seen as critical for organisational uptake. Specific suggestions included allowing workplaces to test the program in advance and clearly outlining its benefits to workplace leaders.

*If you were to demonstrate the positives of a healthier and fitter, mentally capable, workforce and empathetic workforce…relating it back to dollar signs whether it be psychosocial risks or…claims and lost days and that from mental health, I think you'd potentially get the ears of the right people.*

*(Male, 26–35 years, employee, manufacturing)*

One manager said it was important to identify Helipad as an *evidence-based program* to compete against other mental health programs and apps and leverage partnerships with external agencies, including Employee Assistance Programs (EAPs) and health insurers. This participant also thought that including custom links to directly access a counsellor or EAP through programs such as this could potentially improve existing models - *“there are other platforms that are trying to do it….[where] you can connect with a counsellor or professional online” (Male, 56–65 years, manager, manufacturing)*, which they believed was particularly useful for rural areas. This same participant also suggested that partnering with private health insurers could make the intervention available to organisations en masse, who would then be *“wrapping it together as part of a broader wellbeing strategy.”* This integration with workplace interventions that support teams rather than individuals may enhance both the effectiveness and implementation of Helipad in workplaces.

## 4 Discussion

The Helipad program was found to be more engaging and suitable for implementation in the workplace than an active control condition, based on participant ratings of accessibility, appropriateness, and feasibility in the context of a cRCT of a single-session digital intervention. Quantitative findings indicated that only the intensity of engagement activities at the workplace level was associated with program uptake, suggesting that the program is likely to have similar levels of usage across diverse workplace settings, with uptake driven by organisational engagement and leadership support. Nevertheless, uptake across workplaces was typically low, with a mean of approximately 10 participants per site, despite most workplaces including between 50 and 200 employees, suggesting considerable barriers to reaching employees who may benefit from the program. This level of uptake suggests that real-world implementation may be challenging without organisational buy-in, although it remains possible that making the program continuously available without requiring completion of research surveys may improve accessibility for those most in need of support.

Quantitative findings also suggested that individual implementation factors differed by demographic and workplace characteristics, with office-based employees rating Helipad higher on appropriateness and feasibility, and adults aged 36–55 years and those with higher levels of education reporting lower levels of acceptability. These findings suggest that digital programs may be easier to access in office-based settings, and that those in mid-life may be less receptive to digital interventions or mental health content than younger or older employees. Similar effects of age were reported by Woodard et al. (2024). They suggested that the peak in other life responsibilities at this age that may prevent engagement.

Greater distress was also associated with lower feasibility ratings for Helipad (Cornish et al., 2019). The program's focus on mental health prevention and promotion may have been perceived as less relevant to people who already had previous engagement with mental health services. It is also possible that employees with limited interest in or perceived need for mental health support may not have engaged with the trial. It should also be noted that effects of individual and workplace factors were typically modest (*p* > .01), suggesting that implementation outcomes may vary only marginally based on these characteristics.

Based on the qualitative implementation evaluation, the Helipad program (CFIR *‘innovation’*) was evaluated as comprehensive, holistic, engaging, and relatively easy to use in qualitative interviews, consistent with other studies evaluating digital programs in workplaces (e.g., (Amsalem et al., 2023)) and supporting its implementation. Factors perceived to facilitate implementation of the program included workplace leaders investing in the program by being convinced of its value, and by modelling the importance of taking care of mental health in the workplace. Accordingly, increased encouragement from management and embedding the program in a learning management system were both found to improve participation rates in the current study.

Several participants noted that once they had started the program, they thought they should finish it, which may reflect a role of conscientiousness particularly among interview volunteers. In the *individuals* domain, personality characteristics such as conscientiousness have been found to impact program engagement and uptake in previous studies of digital mental health programs (Gulliver et al., 2021). In our efficacy study (Batterham et al., n.d.), Helipad was more effective at achieving changes in intentions to seek help from professionals than the control program. However, as recognised by several participants in the current study, its effectiveness is likely to depend on successful implementation, and particularly in engaging those who have no experience, knowledge or interest in mental health, which remains a challenge in research and implementation (Gulliver et al., 2025, Gulliver et al., 2020). Promotion of help-seeking interventions in workplaces may be a key prevention strategy, supporting diverse adults to gain the skills and attitudes to engage with services before they experience high distress (Kelloway et al., 2023).

Establishing a positive workplace culture from leadership and employees was seen as a critical driver of implementation in the *inner setting* domain. Participation rates depended strongly on whether managers endorsed the program by having additional meetings to discuss, sending additional reminders encouraging workers to participate, or inclusion in the workplace learning management system. These strategies, also relevant to the *implementation process* domain, all appeared to be effective strategies to increase employee uptake. Echoing previous findings (Paterson et al., 2024; Yarker et al., 2022), workplace endorsement at higher managerial levels, including allocated time during work hours and embedding the program into existing learning management systems, were viewed as facilitating implementation (Bernard et al., 2022). A workplace culture that prioritises mental health has been found to be important for increasing engagement in workplace programs that promote access to professional help (Woodard et al., 2024). The interviews indicated the importance of leadership that understands the organisational benefits of participating in such programs. As a strategy relevant to the *outer setting*, emphasising potential of programs to address the costs of untreated mental disorders in the workplace, as we did in our recruitment letters, and the prevention of workplace psychosocial hazards outlined in legislation (Work Health and Safety, 2024) may also be beneficial.

### 4.1 Implications and recommendations

The current study suggests several strategies for implementing workplace mental health programs and increasing engagement. Strategies to enhance program delivery in the workplace could include clarifying the expected time needed to complete the program and allowing employees to do so during paid work hours with sufficient time and privacy. It is also important to clearly outline the program's purpose and potential benefits to increase buy-in from organisational leaders. The goal of the program to increase help seeking and reduce stigma should be emphasised during roll-out, although blinding limited how much we could describe the primary outcomes in this trial. Partnering with agencies (e.g., EAP providers, insurers, industry bodies) that engage multiple workplaces across diverse industries may also enhance ongoing implementation. The requirement of workplaces to have available EAP services was both a safety consideration and a strength in this trial, as it ensured that all employees had accessible low-intensity support when needed, which could facilitate more intensive support. Implementing Helipad into a holistic EAP service may provide more seamless access to support, while noting that some interviewees reported concerns about the breadth and quality of current EAP services. It may also be helpful to combine individual-focused interventions with organisational and manager-focused strategies to support team mental health, which could enhance implementation compared to delivering individual-focused interventions alone. Workplace digital mental health interventions should also be designed with simplified language, clean minimalistic design, sufficient (but not too much) interactivity, and readable text size.

### 4.2 Limitations and strengths

The key strength of the study is that it provides a comprehensive assessment of implementation outcomes for a workplace mental health intervention, in a trial incorporating diverse industries and using both qualitative and quantitative approaches. However, the current findings may not be generalisable to international workplaces or other programs. Comparative interviews with participants in the active control condition may have elucidated the factors that are general versus specific to the implementation of the Helipad program. Therapeutic interventions or in-depth mental health programs that require engagement over multiple sessions may have different drivers of implementation. Response biases such as social desirability may have influenced participant ratings. Further, those most interested or with pre-existing positive views may have been more likely to participate in both the cRCT and the interviews (selection bias), which may have been influenced by the remuneration for an interview. In particular, a majority of the sample was female and highly educated, with under-representation of industries where stigma reduction may potentially have greater impact. These issues of representation are inherent in research of this type, with ongoing challenges in appealing to uninterested individuals and those who are already engaged in treatment (Gulliver et al., 2025; Ocloo and Matthews, 2016). We did not collect data on whether participants were working remotely, on-site, or a hybrid, although there were no exclusions for off-site workers to maximise ecological validity. Further examination of specific implementation barriers for off-site workers may be needed particularly in highly dispersed or decentralised workplaces. Regression analyses explained limited variance, suggesting implementation outcomes were consistent across sociodemographic groups and unmeasured motivational factors may better explain variations. Finally, we did not collect data on additional implementation outcomes such as fidelity and sustainability.

## 5 Conclusion

The Helipad program was viewed as an acceptable, feasible and appropriate workplace program for the promotion of mental health help seeking. Recommendations for optimising the implementation of workplace mental health programs include establishing partnerships with agencies such as health insurers or industry bodies. In addition, implementation may be enhanced by clear communication of the benefits of the program to workplace leaders to ensure organisational commitment, and the integration of individual-level programs with other workplace interventions including those targeting managers or teams.

## CRediT authorship contribution statement

Conceptualization: PB

Methodology: PB, AG

Formal analysis: PB, AG, CH

Investigation: PB, AG, CH

Resources: PB

Data Curation: AG

Writing - Original Draft: PB, AG, CH

Writing - Review & Editing: AW-S, AT, LF, ALC, MB

Visualization: AG

Supervision: PB

Project administration: PB, AG, CH

Funding acquisition: MB, PB, ALC, AT

## Consent for publication

Not applicable.

## Ethics approval and consent to participate

All participants provided informed consent online. The research was approved by The Australian National University Human Research Ethics Committee (ANU HREC protocol number 2023/053).

## Funding

This trial was supported by the 10.13039/501100006623Mental Health Australia General Clinical Trials Network (MAGNET was funded by a peer-reviewed 2020 MRFF Million Minds Mission MHR grant (MRF2006296). The lead author can disseminate the results of this trial without the express permission of the funder. AW-S is supported by a 10.13039/501100000925NHMRC Emerging Leader Investigator Grant (1197074). MB is supported by a NHMRC Senior Principal Research Fellowship and Leadership 3 Investigator grant (1156072 and 2017131).

## Declaration of competing interest

Authors PB, AG and CH were involved in the development of the Helipad program (https://helipad.net.au, 2024) although they receive no personal financial gain from the program.

## Data Availability

De-identified data will be retained for data aggregation purposes (e.g., meta-analyses) and data sharing. Data will be archived for use in future research with permission from the Principal Investigator (PJB) or his nominated delegate. Data will be shared upon reasonable request to the Principal Investigator (PJB) or his nominated delegate. The de-identified data will be made available for research purposes through data repositories including HeSANDA (Health Studies Australian National Data Asset) and ANU Data Commons.

## Appendix A Supplementary data

Additional file 1 CONSORT checklist

Additional file 2 Recruitment method examples

Additional file 3 Participant flow diagram

Additional file 4 Interview questions

Additional file 5 Regression model for participation rates (uptake)

Additional file 6 Detailed interview feedback on Helipad program
