# Supplementary material for: Determinants of implementation success for a digital single-session intervention for workplace mental health: Mixed methods evaluation in a cluster trial
Source: Internet Interv. 2026 Jun 23;45:100970. doi: 10.1016/j.invent.2026.100970 (PMC13316626; doi:10.1016/j.invent.2026.100970)
Supplement: Additional file 2 — Recruitment method examples [file mmc2.docx]

# Additional file 2- Recruitment method examples

**Box 1.1.** Standard recruitment email invitation sent from workplaces to employees

*Dear <employees>,*

*We would like to invite you to participate in a voluntary research study evaluating an online program designed to change knowledge and beliefs around mental health in workplaces.*

*The study will take approximately 40 minutes to complete. This includes a short survey (10 mins), followed by a brief program (20 mins), and another survey (10 mins) to check up on how you’re doing after completing the program.*

*Please click the <link> or access the QR code below to participate.*

*<QR code>*

*Best wishes,*

*<workplace name>*


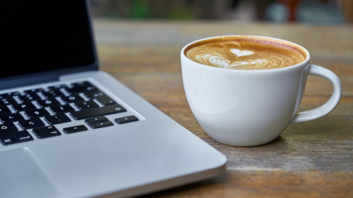


*On behalf of*

*The HeLiPaD project team*

*E:* [*helipad@anu.edu.au*](mailto:helipad@anu.edu.au)

*This study is conducted by researchers from The Australian National University (ANU), Black Dog Institute, and Deakin University and is supported by funding from the Mental Health Australia General Clinical Trials Network (#MRF2006296). The ethical aspects of this research have been approved by the ANU Human Research Ethics Committee (2023/053).*


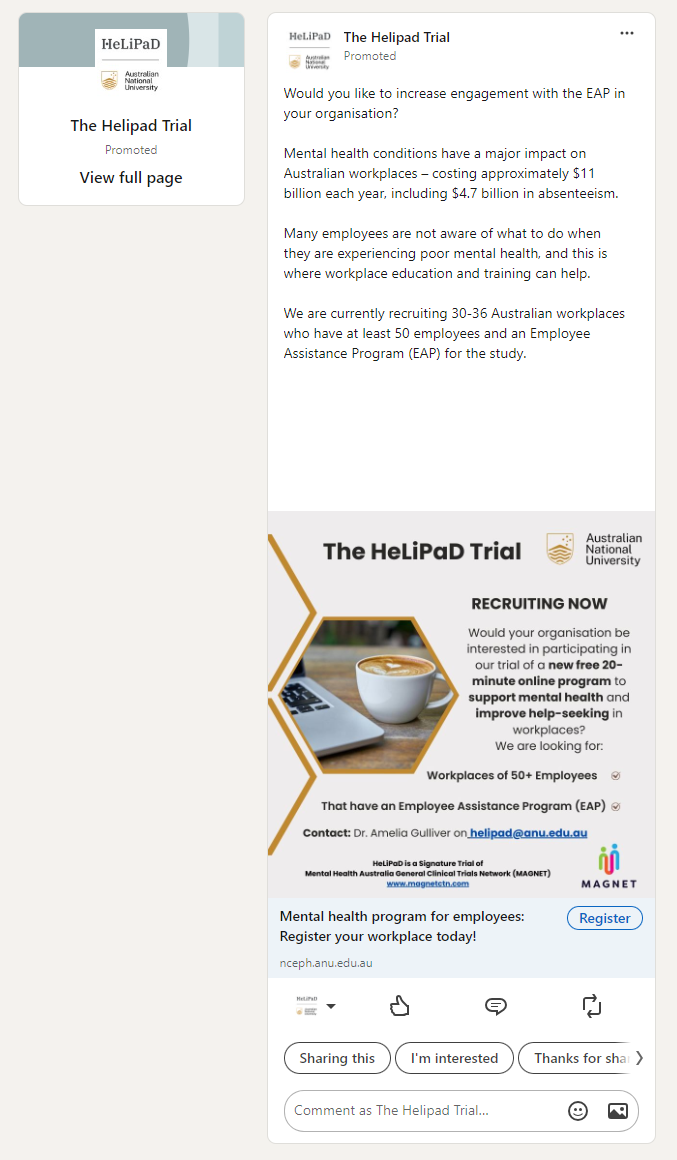


**Figure 1.1.** LinkedIn advertising example
