# Supplementary material for: Determinants of implementation success for a digital single-session intervention for workplace mental health: Mixed methods evaluation in a cluster trial
Source: Internet Interv. 2026 Jun 23;45:100970. doi: 10.1016/j.invent.2026.100970 (PMC13316626; doi:10.1016/j.invent.2026.100970)
Supplement: Additional file 3 — Participant flow diagram [file mmc3.docx]

**Additional file 3 -** Participant flow diagram

Figure 2.1 shows the participant flow through the trial. During May 2024 to January 2025, we recruited a total of 487 (control=264; intervention=223) participants across 51 randomised eligible workplaces (control=26; intervention=25).


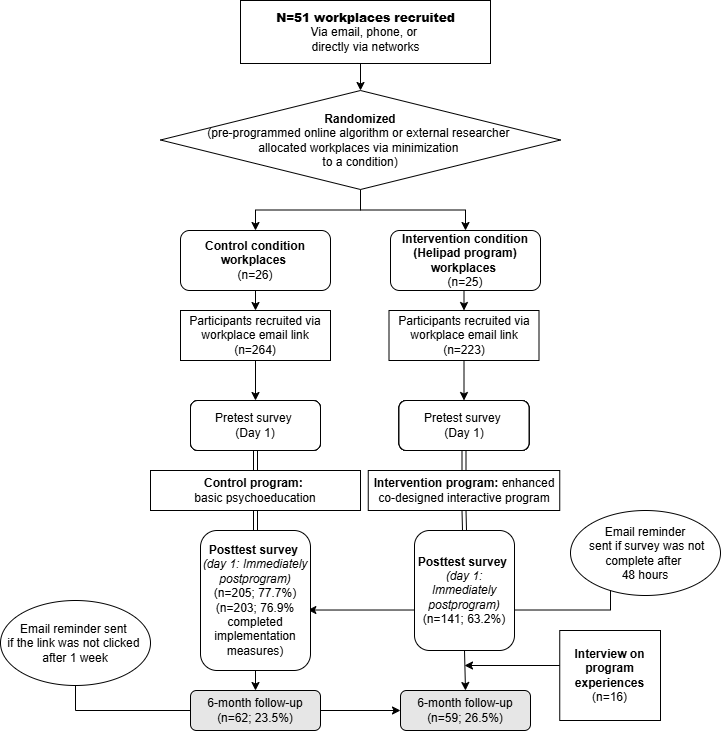


Figure 2.1. Cluster randomised controlled trial flow chart
