# Supplementary material for: Determinants of implementation success for a digital single-session intervention for workplace mental health: Mixed methods evaluation in a cluster trial
Source: Internet Interv. 2026 Jun 23;45:100970. doi: 10.1016/j.invent.2026.100970 (PMC13316626; doi:10.1016/j.invent.2026.100970)
Supplement: Additional file 4 — Interview questions [file mmc4.docx]

**Additional File 4 -** Interview questions

**Box 3.1.** Interview questions

*1. Tell me about your general experience of the program? Prompt: Were you involved in the roll-out of the program (i.e., as management)? (If yes, is there anything you wanted to add about that experience, challenges?)*

*2. Which parts of the program did you use most, and why? Prompt: e.g., Which page, or which tools?*

*3. What did you like, or what was helpful about the program?*

*4. What do you think you might improve about it if you could? Prompts: anything about the content, length, or the design, delivery format of the program?*

*5. How did you hear about the program and what motivated you to try it out?”*

*6. How might we encourage other workplaces or individuals to try the program? Prompts: e.g., what types of structures around it would help with engagement such as getting management onboard, incentives etc*

*7. Did you complete the program? Why or why not?*

*8. Is there anything else you wished to add about the program?*
