# Supplementary material for: Determinants of implementation success for a digital single-session intervention for workplace mental health: Mixed methods evaluation in a cluster trial
Source: Internet Interv. 2026 Jun 23;45:100970. doi: 10.1016/j.invent.2026.100970 (PMC13316626; doi:10.1016/j.invent.2026.100970)
Supplement: Additional file 5 — Regression model for participation rates (uptake) [file mmc5.docx]

**Additional file 5 –** Regression model for participation rates (uptake)

Participation rates in percentages by method of encouraging uptake used for the workplace sites coded as were *Minimal* (*M*=3.8%; *SD*=4.0%); *Some* (*M*=8.1%; *SD*=6.5%); and *Comprehensive* (*M*=9.21%; *SD*=3.17%). As noted in the manuscript, compared with minimal encouragement (e.g., standard emails or newsletter alone), both *some* encouragement (e.g.,1-2 methods; *p*=.006) and *comprehensive* encouragement (e.g., additionally embedding the program in a learning management system; *p*=.041), were significant predictors of increased participation rates (uptake).

**Table 4.1.** Linear regression model for participant uptake (%) predicted by workplace characteristics (*N*=344)

| *Participant uptake (%)* | | | | |
| --- | --- | --- | --- | --- |
|  | *Estimate* | *SE* | *t* | *p* |
| Intercept | 2.380 | 2.572 | 0.925 | .360 |
| *Some* (1-2 additional methods i.e., pre-emptive email, mentioned in meeting)**^b^** | 4.481 | 1.567 | 2.860 | **.006^a^** |
| *Comprehensive* (embedded in LMS + multiple methods, reminders)**^b^** | 5.309 | 2.520 | 2.107 | **.041^a^** |
| Workplace type (Office-based) | -2.209 | 1.488 | -1.484 | .145 |
| Organisation size (Large ≥200) | 2.862 | 1.520 | 1.883 | .066 |
| Location (Regional) | 0.047 | 2.143 | 0.022 | .983 |
| Condition (Helipad) | 0.550 | 1.392 | 0.395 | .695 |

***Note:*  ^a^ p<.05.** Reference group is presented in (brackets); **^b^** (vs Minimal encouragement).
