# Supplementary material for: Determinants of implementation success for a digital single-session intervention for workplace mental health: Mixed methods evaluation in a cluster trial
Source: Internet Interv. 2026 Jun 23;45:100970. doi: 10.1016/j.invent.2026.100970 (PMC13316626; doi:10.1016/j.invent.2026.100970)
Supplement: Additional file 6 — Detailed interview feedback on Helipad program [file mmc6.docx]

**Additional file 6 -** Detailed interview feedback on Helipad program

***Content and functionality***

Further feedback indicated the participants liked the examples and videos of people sharing their experiences, indicating that the content normalised mental health and helped to enable conversations. However, one participant remarked it might be important to add *“an example where like things maybe don't go smoothly, but the outcome is still good and kind of provide some strategies”* for managing that situation *(Participant 4, employee, public services)*. Multiple participants praised the hypothetical scenarios that described how to respond to people disclosing their mental ill-health in the workplace to managers and colleagues. This was primarily because they offered scripts, as considered not knowing what to say, particularly in the workplace was a *“massive barrier”* and these examples *“might really help people and reduce that fear of having those conversations.” (Participant 15, manager, education).* When asked what was missing from the Helipad program, one participant said “*the obvious one is around, perhaps people with a different cultural background. And there's always a cultural overlay in reaching out for support.” (Participant 1, manager, manufacturing).*

Regarding the functionality and look and feel of Helipad, views differed between participants. Some felt that it was well-ordered and structured, *“straightforward and easy to use” (Participant 10, manager)*, with a *“clean and minimalistic”* visual interface with a good layout that made it more engaging and *“easy to navigate” (Participant 3, employee, public services).* Most participants praised the interactivity of the Helipad program; although some remarked that there was too much, and *“a lot of different things to kind of click and read” (Participant 4, employee, public services)*. Several participants noted that it may be most likely to be useful to those without experience of mental ill-health, although depending on individual needs, it was possible could skip information that was already known. However, others observed there was a lot of detail and information – *“I found it like quite digestible…[but] there was just there was a lot to kind of go through*” *(Participant 4, employee, public services)*, and that the level of detail on different options may confuse or overwhelm people, particularly if they are currently unwell – *“Sometimes I think if we give a lot of information, we make it look complicated.” (Participant 12, employee, health).* Potentially due to this, some reported that it took longer than they expected. Suggestions on how to improve this included making it punchier and more concise and clarifying ‘basic’ content vs additional information, and that the links to other resources were optional, particularly for workplaces where employees may be time poor. Overall, offering multiple delivery modes (e.g., written, audio, visual) to cater to different learning styles, and situations (e.g., in public places where sound is inaccessible) was suggested to allow people to engage flexibly.

***Module feedback***

Table 4.1 presents a full list of the feedback collected in the interviews. Feedback on specific modules included that Module 1 on recognising symptoms was a great starting point in helping employees in recognising and *“normalising…feelings and behaviors” (Participant 11)* particularly for understanding the link between physical and psychological symptoms and identifying burnout. It was also deemed particularly useful for people less knowledgeable about mental health, such as *“if someone’s experiencing issues, then they don’t know where to start” (Participant 8).* Module 2 on getting support was generally thought to provide clear pathways to care, and address *“preconceived ide*as” (Participant 6) about mental health professionals that may not be accurate and which may help to build trust in professionals. However, there were some issues with ease of interpretation because of the way it was presented visually (i.e., arrows were too small to see clearly). Module 3 on treatment options was regarded as being comprehensive on the topic and potentially help to reduce fear around treatments, by helping people understand how different types options work (e.g., cognitive behaviour therapy). However, it was noted as being less interactive, and perhaps overwhelming to process given the amount of information presented. Many participants enjoyed the practical nature of Module 4 on helping others and found the scenarios and scripts helpful by reducing fear around these conversations and showing how different reactions can impact those who disclose. Adding information about what the employee could do next in the scenario was suggested as a way to improve this module. Finally, feedback for Module 5 on creating supportive workplaces included praise for the lived experience videos, information targeting specific concerns about disclosure, and quiz that may have helped to improve understanding; however, one person thought the quiz content was a bit repetitive. In addition, given it was the last module, some people skipped over this content quickly.

**Table 4.1.** Detailed summary of participant feedback on Helipad modules

| Module | Summary of feedback |
| --- | --- |
| **Module 1:**  Recognising symptoms | - A few felt this was the standout module (Participant 10, 11). - *‘Because I think if someone’s experiencing issues, then they don’t know where to start, that’s a really good starting point.’* (Participant 8). - Good visual representation - would be also useful as poster/visual aid for employees to use daily (Participant 10). - Zones were useful to help recognise what person was feeling and what to do - normalising (Participant 11). - Prompts you to check-in with yourself. - Helpful to recognise what is normal or not and connection between mental and physical reactions (Participant 4). - Helpful to recognise burnout, which might not be as apparent as other mental health crises. |
| Module 2: Getting support | - Good alternative to social media as a source to remember how/where to get help - Good for people struggling to know where to go (Participant 8), and literacy around help-seeking. - Good explanations of different health professionals - eliminate preconceptions (Participant 6) and build trust in services such as EAP (Participant 10). - Not as helpful to those who know where to get support, but potentially useful for those who are not aware (Participant 4) - Helpful for those who are supporting others to know where to direct them - Good mix of words, visual and interactive. - Text could be larger or easier to read. - Pathway was confusing/difficult to follow - arrows too small and Step 1/1A not clear. - Questions more useful for someone with low literacy or who did not know what to ask. |
| Module 3: Treatment options | - Very comprehensive and accessible, but not overly technical (Participant 9) - Neatly summarised and one participant stated this was the most informative module. - Information not as necessary until needed- might be overwhelming for someone new to process (Participant 4). - Some only read what they were interested in or unaware of.   Helpful to know there are different options (other than medication) - may not have considered or heard of (Participant 8, Participant 9).   - Felt could consider all options equally. - May be useful information for support person (Participant 4). - Clearly explains CBT (Participant 1). - CBT diagram takes fear out of unknown- more user-friendly (Participant 8). - CBT diagram or information may be useful as poster or fact sheets - reinforcement in conjunction with other MH training in workplace (Participant 10). - One participant said support people not as helpful, but may be a useful reminder (Participant 4); another participant said helpful in showing different people to seek help from. - Could be more interactive (Participant 8). - Filter functionality was not obvious. - Needs to be more obvious that person can click-through diagrams (i.e., click image of support persons) (Participant 10). |
| Module 4: Helping others | - Hypothetical situations more helpful as more practical. - Useful to see what you should not do in comparison and liked that information was specific to workplace (Participant 8). - Scripts reduce the fear/barrier to these conversations. - ‘Depersonalised’ and scenarios only show positive outcome - good to have worst-case scenario and how to navigate/coping strategies (Participant 4). - One participant found these examples useful as similar examples not easy to come by (e.g., internet searching) and encourages people to seek help and managers to support staff (Participant 6). - Would be useful/valuable for management/leadership to do (Participant 10). - Useful for vulnerable, disclosing employee to see what it’s like to get support (Participant 10). - Could use some text around what to do next for employee (Participant 10). - Would be useful to see part of response for each scenario. |
| Module 5: Supportive workplaces | - Felt too general - depends on workplace/scenario (Participant 4). - Good amount of interactivity (Participant 10). - Lived experience videos were thought to help to reduce stigma with real stories. - Quiz was useful to go over content and ensure understanding (Participant 5), but some felt it was a bit repetitive (Participant 7). - ‘My concerns’ section explaining specific concerns people may have was helpful depending on workplace culture - still useful as stigma still exists even in good cultures. - Some went over this module quickly due to time constraints. |
| Videos | - Most participants especially liked the videos. - Lived experience videos particularly resonated - positive workplaces, but one participant remarked the content was overly positive (Participant 4), whilst another remarked they were too simplistic- would be useful to alleviate fear to go over a more difficult scenario and strategies *‘seeing an example where everything goes right, I think, sometimes doesn’t resonate really quite the same way.’* (Participant 4). - Some participants would have preferred more videos (Participant 9), particularly around lived experience (Participant 2) “*I just think the fact that there are people there talking about their experiences really, really valuable”* (Participant 9) - One participant suggested having lived experience videos that included tips on how to improve mental health at work - Helpful to get different perspectives (Participant 5). - Video scenarios useful to those without experience (Participant 10). - Not too long. - Some videos seemed ‘forced’, particularly manager video. - 1 participant didn’t really view the videos (prefers reading) (Participant 4). - Good to have subtitles, in case person needs to mute due to external situation (Participant 6). - Videos did not run automatically and were on a continuous loop (this was annoying)- autoplay of next video would be more helpful. |
